# Relationship between Job Stress and 5-HT2A Receptor Polymorphisms on Self-Reported Sleep Quality in Physicians in Urumqi (Xinjiang, China): A Cross-Sectional Study

**DOI:** 10.3390/ijerph15051034

**Published:** 2018-05-21

**Authors:** Xiaoyan Gao, Hua Ge, Yu Jiang, Yulong Lian, Chen Zhang, Jiwen Liu

**Affiliations:** 1 Department of Public Health, Xinjiang Medical University, Urumqi 830011, China; 15199142607@163.com (X.G.); gehua2710@sina.com (H.G.); 2 Department of Public Health and Management, Wenzhou Medical University, Wenzhou 325000, China; yuyu_jiang88@126.com; 3 Department of Public Health, Nantong University, Nantong 226000, China; lianyulong444@163.com; 4 Hospital Management Office, Xinjiang Medical University, Urumqi 830011, China

**Keywords:** physicians, job stress, 5-HT2A, sleep quality

## Abstract

The serotonin receptor (5-HTR) plays a key role in sleep quality regulation. Job-related stress is an important factor that influences sleep quality. However, few reports on the interaction between 5-HTR2A polymorphisms and job stress, and how they may impact upon sleep quality are available. Therefore this study investigated the effects of job stress, 5-HTR2A polymorphisms, and their interaction on sleep quality, in physicians. Using a two-stage stratified sampling method, 918 participants were initially invited to participate in the study. After screening for study inclusion and exclusion criteria, 504 subjects were eventually included in the study. Job stress and sleep quality were assessed using the Job Stress Survey (JSS) and Pittsburgh Sleep Quality Index (PSQI), respectively. The 5-HTR2A receptor gene polymorphisms T102C and -1438G/A of were determined using polymerase chain reaction-restriction fragment length polymorphism. Job stress was significantly associated with sleep quality. High levels of job stress were linked to a higher risk of poor sleep quality compared to low or moderate levels [odds ratio (OR) = 2.909, 95% confidence interval (CI): 1.697–4.986]. High levels of stress may reduce subjects’ sleep quality, leading to an increase the likelihood of sleep disturbances and subsequent daytime dysfunction. The 5-HTR2A receptor gene polymorphism T102C was not significantly associated with sleep quality in this study, however, the -1438G/A polymorphism was significantly associated with sleep quality. The GG genotype of the -1438G/A polymorphism was linked to poorer sleep quality. When compared with subjects with low job-related stress levels×AG/AA genotype (OR = 2.106, 95% CI: 1.278–3.471), physicians with high job-related stress levels×GG genotype had a higher risk of experiencing poor sleep quality (OR = 13.400, 95% CI: 3.143–57.137). The findings of our study indicate that job stress and 5-HTR2A receptor gene polymorphisms are associated with sleep quality in physicians. Subjects with high job stress level or/and the -1438G/A GG genotype were more likely to report poor sleep quality, and furthermore, their combination effect on sleep quality was higher than their independent effects, so it may be suggested that job-related stress and genes have a cumulative effect on sleep quality; that is, stress can increase the risk of poor sleep quality, but this effect is worse in a group of people with specific gene polymorphisms.

## 1. Introduction

Sleeping and awakening are essential physiological processes that are necessary for human survival. Good sleep quality helps to maintain good wakefulness. However, more and more people complain about experiencing poor sleep quality. Estimated rates of insomnia vary between 6% and 48%, depending on the definition and sample or country [1,2,3]. Recent studies have shown that poor sleep quality is associated with psychological problems [4,5,6], physiological and chronic diseases [7,8,9], and an increased risk of non-fatal and fatal occupational injuries [10,11]. These are a great burden to the individual and are a significant cost to society [12,13,14]. Sleep problems are currently one of the leading health problems.

Many factors can influence sleep quality, including demographics, social support, health status, sleeping environment, and work and life-related stressors [15]. The influence of work-related stress on the physical and mental health of populations from different occupations recently become of great interest. Some studies have demonstrated the relationship between insomnia and various types on occupational stress, such as job demand, job control, social support, job insecurity, organizational justice, intragroup conflict, job strain, effort–reward imbalance, employment level, and shift work [16]. A group of Japanese scholars have published on the relationship between job stress and sleep quality, and the results suggest that job stress is indeed associated with sleep quality, and that it increases the risk of experiencing sleep problems [17,18,19,20,21,22,23]. Studies from other countries have also found similar relationships between sleep and work-related stress [24,25,26,27,28,29,30]. Furthermore, a number of studies have indicated that sleep deprivation can increase allostatic responses to psychosocial stress, but daytime sleepiness is associated with a reduced response to stress [31,32]. These results all suggest a strong link between sleep and work-related stress.

In addition to job stress, genetic factors may also increase the risk of sleep problems. Twin and family studies have indicated that circadian rhythms are not learned, but are the result, in part, of multiple heritable influences [33,34]. Over the years, researchers have been looking for genetic information pertaining to sleep. Animal and human studies have shown that sleep is associated with many neurotransmitters in the brain. Serotonin (5-hydroxytryptamine, 5-HT) is a key central nervous system neurotransmitter that regulates numerous physiological functions, including appetite, thermoregulation, pain perception and hormone secretion, etc. [35]. Additionally, 5-HT has been shown to be related to the occurrence and maintenance of sleep [36,37,38]. The various physiological functions of 5-HT are mediated via seven distinct receptor families (5-HT1–5-HT7). Studies have found that the 5-HT2A receptor is highly enriched in the medulla oblongata, dorsal raphe nucleus, hippocampus, cerebral cortex, and that these structures are relevant to sleep [39]. Meanwhile, genetic association studies of the 5-HT2A gene have revealed a very strong linkage disequilibrium of the -1438G/A polymorphism with the T102C polymorphism [40]. Relationships between the -1438G/A and/or T102C single nucleotide polymorphisms and risk of sleep problems have been found in obstructive sleep apnea (OSA) [41], sleep bruxism [42], and in some psychological problems, which accompany sleep disturbance [43].

Previous studies have highlighted the roles of work-related stress and 5-HT receptor genes in sleep quality, respectively. However, the relationship between these three parameters has rarely been the focus of previous research. Therefore, we carried out a study to examine the independent and interactive effects of 5-HTR2A gene polymorphisms and job stress on sleep quality amongst physicians in the Urumqi of Xinjiang Province, China.

## 2. Materials and Methods

### 2.1. Subjects

This study was carried out between March 2015 and July 2016. All subjects gave their informed consent before they participated in the study. The study was conducted in accordance with the Declaration of Helsinki, and the protocol was approved by the Ethics Committee of Xinjiang Medical University (2015006). The study subjects were physicians working in six hospitals affiliated with Xinjiang Medical University in the Urumqi of Xinjiang Province, China. The six hospitals comprised of four general hospitals and two specialized hospital. For the study, we randomly selected two general hospitals and one specialized hospital. In total, 1836 physicians were working in these three hospitals. As a large number of blood samples and questionnaires needed to be collected, we applied computer-generated random numbers to the managers’ list of employees from each hospital to select 50% (918) of the participants. We conducted an interview, collecting sociodemographic data, job stress, and sleep quality status, and collected blood samples. The inclusion criteria were as follows: (1) physicians who have been working for >1 year and were aged between 20 and 60 years; and (2) voluntary consent to participate after being informed of the objective and significance of the study (i.e., informed consent) was obtained. The exclusion criteria were as follows: (1) a previous diagnosis of chronic and/or other diseases that may affect sleep quality; (2) use of any medication in the three months prior to the study which may affect the serotonergic system and/or sleep/wake cycle; (3) refusal to participate in the study or being stationed abroad; and (4) incomplete collection of relevant information and/or the absence of a blood sample. Subsequently, 504 physicians were included in the study. Each participant was compensated with RMB 30 Yuan for breakfast in this study.

### 2.2. Measures

#### 2.2.1. Sleep Quality

Subjective sleep quality was assessed using the Pittsburgh Sleep Quality Index (PSQI). This scale was developed by Dr. Buysse, a psychiatrist at the University of Pittsburgh in 1989 [44]. This scale is validated for evaluating sleep quality in patients with sleep and psychiatric disorders, as well as in healthy individuals. The scale is composed of 19 items within seven categories: (1) subjective sleep quality; (2) sleep latency; (3) sleep duration; (4) sleep efficiency; (5) sleep disturbances; (6) hypnotic use; and (7) daytime dysfunction. Each category is assigned a score from 0 to 3 using a four-point Likert scale, with the total score ranging from 0 to 21 points. A total score cut-off value of 5 points was used [45]. A score ≥5 was classified as poor sleep quality, with higher scores reflecting poorer sleep quality. A score <5 was classified as good sleep quality.

#### 2.2.2. Job Stress

Job stress was assessed using the Job Stress Survey (JSS). This scale was developed by Spielberger et al. in 1986 [46]. It measures the intensity and frequency of stress factors to comprehensively evaluate the stress levels of subjects. The scales’ reliability and validity has been confirmed by other researchers [47,48]. The scale was introduced to China by Lian et al. [49] after obtaining consent from Spielberger et al. The scale was then translated by experts at the Foreign Languages Department to ensure translational accuracy. Under the guidance of experts, relevant economic and cultural items were revised to make the scale more suitable for Chinese physicians. The scale has 60 items. The first 30 items are used to assess the intensity of stress factors, and each item is assigned a score from 1 to 9. The next 30 items are used to assess the number of days of stress experienced over the past 6 months, and again each item is assigned a score from 0 to 9 (with more than 9 days of stress given a score of 9+). The job stress index was the product of the overall intensity and frequency of job-related stress experienced, with higher scores associated with a high level of occupational stress. The quartile method was subsequently used to divide job stress into three categories: (1) low; (2) moderate; and (3) high levels of job stress.

### 2.3. Genotyping

Blood samples were collected into ethylene diamine tetraacetic acid tubes during the physicians’ physical examination. Genomic DNA (gDNA) was extracted using the Whole Blood Genome Extraction kit (Tiangen Biotech, Beijing, China) before cryopreservation at −20 °C until further use. The T102C and -1438G/A polymorphisms were genotyped using polymerase chain reaction-restriction fragment length polymorphism (PCR-RFLP). gDNA (100 ng) was used in each reaction mixture, with the final volume of each reaction mixture totaling 20 μL. gDNA was then amplified using the PCR instrument (MyCycler, Bio-Rad, Hercules, CA, USA). Ten μL of PCR product was then used in each enzyme-digested mixture, with the final volume of each enzyme-digested mixture totaling 30 μL. The fragments were resolved with electrophoresis on 2% agarose gels and visualized with UV light. The primers used and genotype information for T102C and -1438G/A are listed in Table 1 and Table 2.

### 2.4. Covariates

The demographic data collected (i.e., age and ethnicity), as well as job and lifestyle factors are considered to impact upon sleep quality. Therefore they could potentially effect the results of this study. The following variables were taken into account: (1) demographic: sex, age (<30, 30–40, 40–50, >50 years), ethnicity (Han and Minority), educational level (college degree and below, bachelor degree and above), marital status (married, unmarried); (2) job: monthly income (≤5000, >5000 yuan), professional title (elementary, intermediate, advanced), job tenure (<10, 10–20, >20 years); (3) lifestyles: smoking (yes or no), alcohol consumption (yes or no) and regular exercise (yes or no).

### 2.5. Quality Control

In an attempt to reduce investigator bias, all investigators were trained so that all investigations were conducted in a unified way. Participants were asked to complete the questionnaires independently and anonymously, within 5 min, after which all questionnaires were reviewed and coded by specialized investigators using EpiData version 3.1 (The EpiData Association, Odense, Denmark) to establish a database. 20% of entries were randomly compared against the original questionnaires to check the accuracy of the final database. The PSQI is considered the international gold standard, so the evaluation criteria is unified. Collection of blood samples was completed by a member of the medical team. All samples were stored at −20 °C in a freezer. All experimental instruments were calibrated to ensure standard operations prior to experimental work. The same manufacturer and batch number of each reagent was used throughout the experiment. This ensured that all experiments were standardized to optimize all procedures, and to avoid measurement bias.

### 2.6. Statistical Analysis

As PSQI scores were not normally distributed, they were analyzed using the Kruskal–Wallis test. If initial results showed significant differences, post hoc pairwise comparisons were analyzed using Mann–Whitney U tests and *p*-values were adjusted with Bonferroni’s correction. Chi-square (*χ*^2^) tests were used to analyze categorical variables [i.e., genotype and Hardy Weinberg Equilibrium (HWE)]. Odds ratios (OR) and 95% confidence intervals (CI) were used to determine the interaction between job-related stress levels and genotype associated with sleep quality using unconditional logistic regression. The interactions between job stress, genotype, and sleep quality were determined with general linear model and crossover analysis. The tests were two-tailed and the significance level was set at *p* < 0.05 or *p* < 0.01. The data were analyzed using SPSS 17.0 (IBM, Armonk, NY, USA).

## 3. Results

### 3.1. Characteristics of Subjects and Their Associations with Sleep Quality

The subjects’ characteristics and their incidence of poor sleep quality are shown in Table 3. 302 (59.92%) subjects reported poor sleep quality. The prevalence of poor sleep quality was different across sex, educational level, job tenure, alcohol consumption and exercise, and these differences were statistically significant (*p* < 0.05). The prevalence of poor sleep quality was higher in females compared to males (62.37% vs. 51.72%), and subjects with a postgraduate and above level of education had a higher prevalence of poor sleep quality compared to subjects with undergraduate and below level of education (62.44% vs. 48.94%). Subjects with a job tenure between 10 and 20 years were more likely to report poor sleep quality compared to subjects with a job tenure <10 or >20 years (72.00% vs. 41.51% vs. 57.58%). Subjects who drank alcohol and who did not exercise regularly were more susceptible to poor sleep quality when compared to those who did not drink alcohol (62.69% vs. 49.02%) and who exercised regularly, respectively (65.73% vs. 52.29%). The other variables examined were not shown to be statistically associated with poor sleep quality (*p* > 0.05).

### 3.2. Distribution of Sleep Quality throughout Different Levels of Job-Related Stress

The distribution of sleep quality across low, moderate and high levels of job stress were significantly different (*p* < 0.05). The risk of poor sleep quality in subjects with high levels of job stress were higher when compared to those who had low levels of job stress (OR = 2.909, 95% CI: 1.697–4.986). This significant association remained after adjusting for confounding factors, including sex, educational level, job tenure, alcohol consumption and exercise (OR = 2.800, 95% CI: 1.597–4.908). Analysis demonstrated that the association between job stress and sleep quality was statistically significant (*p* < 0.05; Table 4).

### 3.3. Comparisons of PSQI Subscores across Different Job-Related Stress Levels

The PSQI subscores of sleep efficiency, sleep disturbances and daytime dysfunction were significantly different across the different job stress levels (*p* < 0.05). Sleep efficiency scores in subjects with high job stress levels were significantly lower than those with moderate job stress levels (*p* < 0.05). Furthermore, sleep disturbance and daytime dysfunction scores in subjects with high job stress levels were significantly higher than those with low and moderate job stress levels (*p* < 0.017; Table 5).

### 3.4. HWE Tests and the Distribution of Sleep Quality across the T102C and -1438G/A Genotypes

The distribution of the T102C and -1438G/A genotypes did not deviate from HWE in all subjects (*p* > 0.05). Sleep quality distributions of three genotypes of T102C and -1438G/A were analyzed using *χ*^2^ test. The results revealed that sleep quality distribution was significantly different across the three -1438G/A genotypes (*p* < 0.05). Similarly, the results of logistic regression analysis showed that the GG genotype increased the risk of poor sleep quality when compared to the AA genotype (OR = 1.955, 95% CI: 1.170–3.264). However, no significant risk for poor sleep quality existed when examining the different T102C genotypes (OR = 1.337, 95% CI: 0.869–2.057; Table 6).

### 3.5. Interaction between Job Stress and the 5-HTR2A -1438G/A Polymorphism on Sleep Quality

**Figure 1:**
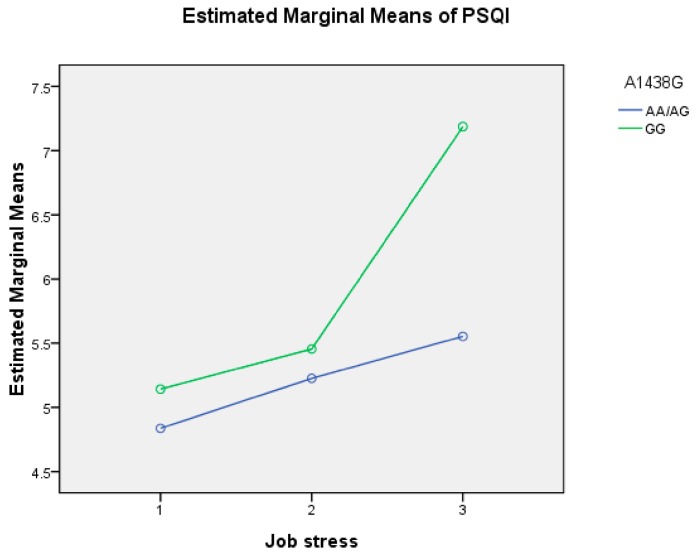
The interaction plot of job stress and -1438G/A on sleep quality.

Job stress was divided into low and moderate, and high levels, and -1438G/A genotypes were divided into GG and AA/AG groups. Job stress and -1438G/A had the main and interactive effects (F_1_ = 9.953, *p* < 0.001; F_2_ = 38.659, *p* = 0.003; F_1__×2_ = 3.403, *p* = 0.034; Figure 1) on sleep quality. The results of crossover analysis showed that subjects with high job stress levels×GG genotype (OR = 13.400, 95% CI: 3.143–57.137) and subjects with high job stress levels×AG/AA genotype (OR = 2.106, 95% CI: 1.278–3.471) were more likely to report poor sleep quality when compared to subjects with low or moderate job stress levels×AA/AG genotype. Statistical significance persisted after adjusting for confounding factors, including sex, educational level, job tenure, alcohol consumption and exercise (OR = 13.707, 95% CI: 3.171–59.261; OR = 2.626, 95% CI: 1.336–3.828; Table 7). The results of stratified logistic regression were also consistent. When stratified according to genotype, there were no significant risks associated with job stress and sleep quality. However, when stratified according to job stress levels, subjects with high job stress and the GG genotype (OR = 15.909, 95% CI: 3.228–78.418) showed a stronger association with poor sleep quality when compared to those with the AG/AA genotype (OR = 2.294; 95% CI: 1.345–3.913, Table 8). The PSQI subscores of subjective sleep quality, sleep latency, sleep efficiency, sleep disturbances, Daytime dysfunction, and total score were significantly different across the different interaction groups (*p* < 0.05). Total score of PSQI in subjects with high job stress levels×GG genotype were significantly higher than those with high job stress levels×AA/AG genotype, low and moderate job stress levels×GG, and low and moderate job stress levels×AA/AG genotypes (*p* < 0.008). Furthermore, subjective sleep quality, sleep latency, sleep efficiency, sleep disturbances, Daytime dysfunction in subjects with high job stress levels×GG genotype were significantly higher than those with high job stress levels×AA/AG genotype, low and moderate job stress levels×GG genotype, or low and moderate job stress levels×AA/AG genotype (*p* < 0.008; Table 9).

## 4. Discussion

In this study, our findings indicate that job-related stress and the 5-HTR2A gene are associated with sleep quality. The subjects with high job stress level or/and the -1438G/A GG genotype were more likely to report poor sleep quality. Furthermore, our results indicate that job-related stress and the -1438G/A GG genotype may have a cumulative effect on sleep quality. These effects were independent of confounding factors that were frequently associated with poor sleep quality, including sociodemographic parameters (i.e., sex and educational level), work (job tenure), and behavioral characteristics (i.e., alcohol consumption and exercise frequency). Our results are consistent with previous studies, which have also demonstrated that genetic polymorphisms, psychosocial and behavior factors impact upon sleep quality [45,50]. Due to the fact that this research is scarce in China, we carried out this research in the Chinese working population.

### 4.1. The Independent Effect of Job Stress and the 5-HTR2A Gene on Physicians’ Sleep Quality

Previous epidemiological studies examining the association between work-related stress and sleep quality have demonstrated that work-related stress is a major factor in disturbing sleep quality [16]. Current evidence suggests that workers exposed to chronic psychological job stressors develop sleep problems as a reaction to this stress [51]. In accordance with this previous research, our study demonstrated that physicians with high job stress levels were at a higher risk of experiencing poor sleep quality than those with low and moderate job stress levels, even after adjusting for confounding factors. Analysis showed that sleep efficiency scores in subjects with high job stress levels were significantly lower than those with moderate job stress levels, and sleep disturbance and daytime dysfunction scores in subjects with high job stress levels were significantly higher than those with low and moderate job stress levels, which indicated that job stress mainly affects these aspects of the physicians’ sleep. A study examining sleep quality amongst health care workers has previously shown that sleep complaints are serious, especially in medical emergency staff [52]. Sleep disturbances can negatively affect work quality and increase the risk of adverse events and safety-compromising behaviors [53].

Additionally, prior research has documented the involvement of central nervous system serotonin (5HT) in sleep regulation, and has indicated that increased 5HT2 receptor stimulation can reduce slow-wave sleep (SWS) [54]. Although the underlying mechanisms are not completely understood, increased activity of serotonergic neurons is associated with wakefulness [55,56]. Therefore, we selected the 5-HTR2A gene polymorphisms T102C and -1438G/A, and examined their effects on sleep quality. The 5-HTR2A receptor gene polymorphism T102C was not significantly associated with sleep quality in this study, however, the -1438G/A polymorphism was significantly associated with sleep quality. The GG genotype of the -1438G/A polymorphism was linked to poorer sleep quality. Results from a recent meta-analysis examining the association between -1438G/A, and T102C single nucleotide polymorphisms and OSA, indicate that the A-1438G, and not T102C, polymorphism of 5-HT2A is a positive risk factor for OSA, especially in males [57]. An animal study has suggested that 5-HTR2A-null mice exhibit longer periods of wakefulness and reduced SWS than their wild-type counterparts [58].

### 4.2. The Interaction between Job Stress and -1438G/A Polymorphism on Sleep Quality

The results of an interaction analysis showed that there was an interaction between job-related stress and -1438G/A polymorphism on sleep quality. The results of a stratified logistic regression showed that subjects with high levels of job-related stress were more likely to report poor sleep quality than those with low and moderate job-related stress levels, which is independent of the A-1438G/A genotype (stratified by genotype in the dominant model, GG vs. AG/AA). However, when participants were stratified according to job stress levels, there were no associations between genotype and sleep quality amongst subjects with high job stress levels. This finding may have two possible explanations; (1) the effects of the GG vs. AG/AA genotype on sleep quality may be masked by alterations in the expression of the -1438G/A locus; or (2) the effect of the -1438G/A polymorphisms on sleep quality is weaker than that of high job stress levels [45]. But the interaction between the -1438G/A genotype and job stress may occur synergistically; that is, the interaction effect (OR = 13.400) is greater than the effect of job stress (OR = 2.106), even when adjusting for confounding factors. Then using the numerical score of sleep quality as the outcome to replicate the results, which also indicated that the PSQI scores of subjects with high job stress level×GG genotype were significantly higher than those with high job stress levels×AA/AG genotype, low and moderate job stress levels×GG genotype, and low and moderate job stress levels×AA/AG genotype, which meant that the subjects with high job stress level×GG genotype were more likely to report poor sleep quality. So these results may be suggested that job-related stress and genes have a cumulative effect on sleep quality; that is, stress can increase the risk of poor sleep quality, but this effect is worse in a group of people with specific gene polymorphisms.

Several lines of evidence suggest that stress, and its related physiological changes, is a possible link between poor sleep quality and adverse health outcomes. Specifically, findings from both animal and human studies have commented on the bidirectional relationship between sleep and the hypothalamic–pituitary–adrenal axis [59,60]. The HPA axis is the bodies’ main neuroendocrine system, responsible for regulating stress responses via modulation of hormones, including cortisol, corticotropin-releasing hormone and adrenocorticotropic hormone [61]. Corticotropin-releasing hormone and adrenocorticotropic hormone infusion have been shown to reduce individuals’ SWS during the sleep and rapid eye movement parts of the sleep cycle, and also increase sleep latency [62], implying that stress interferes with normal sleep via HPA axis activation. 5-HT is a widely distributed monoamine that has been involved in almost every brain function. Stress influences 5-HT activity, an interaction that could be involved in the development of stress-related illnesses. Conversely, serotonergic drugs modulate activity of the HPA axis and physiological responses to stress [63]. 5-HT activity has been shown to be related to stress and to the sleep cycle [64]. Genetics can predict 5-HT system activation, and the 5-HTR2A is the main receptor involved in the mediation of 5-HT stimulation on the HPA axis [65]. Finally, if we could quantify 5-HT levels in the study participants, and examine the relationship between job stress, 5-HT and 5-HT2A receptor gene polymorphisms on sleep quality, the results would be more robust.

### 4.3. Study Limitations

On the basis of previous studies, we carried out a cross-sectional study to examine the effects of job stress and the 5-HT2A receptor gene on sleep quality. Our results indicate that job stress and -1438G/A genotypes are associated with an increased risk of poor sleep quality. However, there are some limitations that should be improved upon in future studies. First, the category “GG- Good sleep quality/High Job stress” had only two cases, giving little meaning to the results of this group. Increases in the numbers of these cases are required to verify the results in future study. Second, sleep quality was only evaluated using subjective questionnaires. An objective measure for it could improve the reliability of these results. Third, some confounding factors related to sleep quality, such as frequency of shift work, stressful life events, etc., were not considered in this study. Fourth, there were a number of difference sources of sample bias in this study, including response bias (e.g., the subjects who had poor sleep quality may have been more willing to complete the study than those who had good sleep quality) and sample selection bias (e.g., physicians tend to work long hours in a stressful environment; people with a genetic predisposition to poor sleep may be less likely to choose this occupation). Finally, it should be noted that good sleep quality was related to alcohol consumption. This result seems unlikely and needs to be further validated. In our study, we only asked subjects whether they consumed alcohol, rather than asking whether they had moderate or excessive alcohol consumption. Other studies have shown that low or moderate levels of alcohol consumption can promote sleep, whilst long-term or excessive drinking can increase the risk of poor sleep quality [66,67].

## 5. Conclusions

This study has demonstrated the relationships between job-related stress, 5-HT2A receptor gene polymorphisms, and sleep quality. Subjects with high job stress level or/and the -1438G/A GG genotype were more likely to report poor sleep quality, and their combination effect on sleep quality was higher than their independent effects. We hypothesize that the 5-HT2A receptor gene and job-related stress may have a cumulative effect on sleep quality. Based on current research results, we plan to carry out a cohort study to verify the relationship between these factors. A previous randomized controlled intervention study has indicated that worktime reduction and an increase in time spent on recovery activities may be beneficial for long-term health and stress [68]. Therefore, we are also considering conducting an intervention study, controlling genetic factors, and reducing job stress levels to observe changes in sleep quality.

## Figures and Tables

**Table 1:** The primers of T102C and -1438G/A.

Genetic Loci.	Primer Direction	Sequence 5′→3′	Amplified Fragment Length
T102C	Forward	TCTGCTACAAGTTCTGGCTT	342 bp
	Reverse	CTGCAGCTTTTTCTCTAGGG	
-1438G/A	Forward	AGCCAGTTCAATGGTGAT	404 bp
	Reverse	ATGTCATAAGCTGCAAGG	

**Table 2:** The genetic loci and genotype information of T102C and -1438G/A.

Genetic Loci.	Enzyme-Digested Fragment Length	Genotype
T102C	342 bp	CC
	216 bp, 126 bp	TT
	342 bp, 216 bp, 126 bp	CT
-1438G/A	404 bp	AA
	251 bp, 153 bp	GG
	404 bp, 251 bp, 153 bp	AG

**Table 3:** Prevalence of poor sleep quality by subject characteristics.

Characteristics	*n*	No. Poor Sleep Quality	Prevalence (%)	*χ^2^* Value	*p* Value
Sex					
Male	116	60	51.72	4.215	0.04
Female	388	242	62.37		
Age (years old)					
<30	34	18	52.94		
30–40	242	144	59.5	1.901	0.593
40–50	154	98	63.64		
>50	74	42	56.76		
Ethnicity					
Han	398	246	61.81	2.81	0.094
Minority	106	56	52.83		
Educational level					
Undergraduate and below	94	46	48.94	5.805	0.016
Postgraduate and above	410	256	62.44		
Marital status					
Unmarried	56	32	57.14	0.202	0.653
Married	448	270	60.27		
Monthly income (yuan)					
≤5000	372	220	59.14		
>5000	132	82	62.12	0.361	0.548
Professional Title					
Elementary	114	64	56.14		
Intermediate	286	180	62.94	2.508	0.285
Advanced	104	58	55.77		
Job tenure					
<10	106	44	41.51		
10–20	200	144	72	27.566	<0.001
>20	198	114	57.58		
Smoking					
Yes	60	34	56.67	0.3	0.584
No	444	268	60.36		
Alcohol consumption					
Yes	402	252	62.69	6.328	0.012
No	102	50	49.02		
Regular exercise					
Yes	218	114	52.29	9.305	0.002
No	286	188	65.73		
Total	504	302	59.92		

**Table 4:** Associations between job stress and sleep quality.

Job Stress	*n*	Good Sleep Quality	Poor Sleep Quality	*χ*^2^ Value	*p* Value	OR (95% CI)	AOR (95% CI)
Low	126	60 (47.62%)	66 (52.38%)			Reference	Reference
Moderate	252	112 (44.44%)	140 (55.56%)	18.87	<0.001	1.136 (0.740–1.745)	0.933 (0.590–1.474)
High	126	30 (23.81%)	96 (76.19%)			2.909 * (1.697–4.986)	2.800 * (1.597–4.908)

Notes: OR, odds ratio; CI, confidence interval; AOR, Adjusted OR for sex, educational level, job tenure, alcohol consumption and exercise; * *p* < 0.001.

**Table 5:** Associations between occupational stress and sleep quality.

Job Stress	*n*	Subjective Sleep Quality	Sleep Latency	Sleep Duration	Sleep Efficiency	Sleep Disturbances	Daytime Dysfunction
Low	126	0.92 ± 0.63	0.90 ± 0.56	0.41 ± 0.61	0.13 ± 0.33	1.22 ± 0.68	1.00 ± 0.62
Moderate	252	1.04 ± 0.60	0.89 ± 0.62	0.51 ± 0.69	0.21 ± 0.44	1.16 ± 0.62	1.17 ± 0.62 ^a^
High	126	1.11 ± 0.72	0.92 ± 0.41	0.40 ± 0.66	0.08 ± 0.27 ^b^	1.46 ± 0.59 ^a,b^	1.52 ± 0.69 ^a,b^
*χ*^2^ value		5.359	1.282	3.095	9.049	20.596	39.112
*p* value		0.069	0.527	0.213	0.011	<0.001	<0.001

Notes: Compared with low job stress levels, ^a^ *p* < 0.017; Compared with moderate job stress levels, ^b^ *p* < 0.017.

**Table 6:** Associations between the T102C and -1438G/A genotypes and sleep quality.

Genotype	*n*	Good Sleep Quality (*n* = 202)	Poor Sleep Quality (*n* = 302)	OR (95% CI)	*p*-Value for OR
T102C					
CC	122	54 (44.26%)	68 (55.74%)	Reference	
CT	264	104 (39.39%)	160 (60.61%)	1.222 (0.791–1.886)	0.366
TT	118	44 (37.28%)	74 (62.71%)	1.336 (0.797–2.239)	0.272
*χ*^2^ and *p*-value		0.439/0.803			
*χ*^2^ and *p*-value for HWE		1.323/0.516			
-1438G/A					
AA	126	60 (47.62%)	66 (52.38%)	Reference	
AG	252	102 (40.48%)	150 (59.52%)	1.337 (0.869–2.057)	0.335
GG	126	40 (31.75%)	86 (68.25%)	1.955 (1.170–3.264)	0.010 *
*χ*^2^ and *p*-value		6.642/0.036 *			
*χ*^2^ and *p*-value for HWE		0.000/1.000			

Notes: OR, odds ratio; CI, confidence interval; HWE, Hardy Weinberg equilibrium; * *p* < 0.05.

**Table 7:** The interaction between job stress and -1438G/A polymorphism on sleep quality.

Job Stress	-1438G/A	Good Sleep Quality	Poor Sleep Quality	OR (95% CI)	AOR (95% CI)
High	GG	2	30	13.400 (3.143–57.137) *	13.707 (3.171–59.261) *
High	AG/AA	28	66	2.106 (1.278–3.471) *	2.626 (1.336–3.828) *
Low and moderate	GG	38	56	1.316 (0.820–2.113)	1.293 (0.792–2.111)
Low and moderate	AG/AA	134	150	Reference	Reference

Note: OR, odds ratio; CI, confidence interval; AOR, Adjusted odds ratio for sex, educational level, number of working years, alcohol consumption and exercise; * p < 0.01.

**Table 8:** Stratified logistic analysis of job stress and -1438G/A polymorphism with respect to sleep quality.

Job Stress	GG		AG/AA		OR (95% CI)
	Good Sleep Quality	Poor Sleep Quality	Good Sleep Quality	Poor Sleep Quality	
High	2	30	28	66	OR_3_ = 4.677 (0.871–25.114)
Low and moderate	38	56	134	150	OR_4_ = 1.283 (0.787–2.091)
	OR_1_ = 15.909 (3.228–78.418) *		OR_2_ = 2.294 (1.345–3.913) *		

Notes: Odds ratios (ORs) were all adjusted for sex, educational level, job tenure, alcohol consumption and exercise. OR_1_, job stress with GG genotype; OR_2_, job stress with AG/AA genotype; OR_3_, genotypes with high level of job stress; OR_4_, genotypes with low and moderate level of job stress. * *p* < 0.01.

**Table 9:** The interaction between job stress and -1438G/A polymorphism on sleep quality.

Group (Job Stress Level-1438G/A Genotype)	*n*	Subjective Sleep Quality	Sleep Latency	Sleep Duration	Sleep Efficiency	Sleep Disturbances	Daytime Dysfunction	Total Score of PSQI
High-GG	32	1.38 ± 0.61 ^a,b,c^	1.13 ± 0.34 ^c^	0.25 ± 0.44	0.25 ± 0.44 ^a^	1.63 ± 0.61 ^b,c^	1.75 ± 0.67 ^b,c^	7.19 ± 1.77 ^a,b,c^
High-AA/AG	94	1.02 ± 0.73	0.85 ± 0.41	0.45 ± 0.71	0.02 ± 0.15	1.40 ± 0.57 ^c^	1.45 ± 0.68 ^b,c^	5.55 ± 1.86
Low and moderate-GG	94	1.00 ± 0.59	0.91 ± 0.58	0.55 ± 0.65	0.26 ± 0.44 ^a,c^	1.26 ± 0.67	1.15 ± 0.59	5.36 ± 2.24
Low and moderate-AA/AG	284	1.00 ± 0.62	0.89 ± 0.61	0.45 ± 0.67	0.15 ± 0.40 ^a^	1.15 ± 0.63	1.10 ± 0.63	5.09 ± 2.40
*χ*^2^ value		11.007	7.971	6.112	22.964	24.164	39.156	29.683
*p* value		0.012	0.047	0.106	<0.001	<0.001	<0.001	<0.001

Notes: Compared with High AA/AG, ^a^ *p* < 0.008; Compared with Low and moderate GG, ^b^ *p* < 0.008; Compared with Low and moderate AA/AG, ^c^ *p* < 0.008.
